# Improved control of *Trialeurodes vaporariorum* using mixture combinations of entomopathogenic fungi and the chemical insecticide spiromesifen

**DOI:** 10.1038/s41598-024-66051-8

**Published:** 2024-07-03

**Authors:** Eleanor L. Dearlove, David Chandler, Steve Edgington, Shaun D. Berry, Gareth Martin, Claus Svendsen, Helen Hesketh

**Affiliations:** 1 UK Centre for Ecology and Hydrology, Maclean Building, Benson Lane, Crowmarsh Gifford, Wallingford, Oxfordshire OX10 8BB UK; 2 RSK ADAS Ltd. ADAS Gleadthorpe, Meden Vale, Mansfield, NG20 9PD UK; 3 Warwick Crop Centre, School of Life Sciences, Wellesbourne Campus, The University of Warwick, Warwick, UK; 4 CABI, Bakeham Lane, Egham, TW20 9TY UK; 5 Certis Biologicals, Columbia, MD USA; 6 BASF Plc, Woolpit, UK

**Keywords:** MixTox model, *Trialeurodes vaporariorum*, Microbial control, Biopesticide, Entomopathogenic fungi, Interactions, Synergy, Antagonism, Additivity, IPM, Microbiology, Fungi, Agroecology, Entomology

## Abstract

Greenhouse whitefly (*Trialeurodes vaporariorum*) is a major global pest, causing direct damage to plants and transmitting viral plant diseases. Management of *T. vaporariorum* is problematic because of widespread pesticide resistance, and many greenhouse growers rely on biological control agents to regulate *T. vaporariorum* populations. However, these are often slow and vary in efficacy, leading to subsequent application of chemical insecticides when pest populations exceed threshold levels. Combining chemical and biological pesticides has great potential but can result in different outcomes, from positive to negative interactions. In this study, we evaluated co-applications of the entomopathogenic fungi (EPF) *Beauveria bassiana* and *Cordyceps farinosa* and the chemical insecticide spiromesifen in laboratory bioassays. Complex interactions between the EPFs and insecticide were described using an ecotoxicological mixtures model, the MixTox analysis. Depending on the EPF and chemical concentrations applied, mixtures resulted in additivity, synergism, or antagonism in terms of total whitefly mortality. Combinations of *B. bassiana* and spiromesifen, compared to single treatments, increased the rate of kill by 5 days. Results indicate the potential for combined applications of EPF and spiromesifen as an effective integrated pest management strategy and demonstrate the applicability of the MixTox model to describe complex mixture interactions.

## Introduction

The greenhouse whitefly (*Trialeurodes vaporariorum*) is a major insect pest causing substantial damage to > 850 plant species, including high-value greenhouse, ornamental and agricultural crops^1^. Damage by *T. vaporariorum* is caused directly by feeding and through the transmission of plant viruses, resulting in crop losses in excess of $1 billion a year^2–4^. The most effective greenhouse insect integrated pest management (IPM) systems are based on preventative applications of arthropod predators and parasitoids^5^ as components of an integrated pest management (IPM) ‘pyramid’ approach^6^. Under this system, IPM-compatible chemical plant protection products (PPPs) are still applied but are used as supplementary treatments to biological control, acting as a second line of defence should pest numbers increase to levels where natural enemies are unable to control them^7^.

Increasingly, IPM practitioners in greenhouse crops are incorporating low-risk plant protection products such as microbial PPPs into their programmes. These biopesticides are typically based on microorganisms such as entomopathogenic bacteria (predominantly *Bacillus thuringiensis*), viruses, fungi, and protozoa^8^. Microbial PPPs for control of *T. vaporariorum* and other species of whiteflies are based primarily on entomopathogenic fungi (EPF). Entomopathogenic fungi infect their host via direct penetration by conidia through the host cuticle and then via proliferation in the host, ultimately killing the insect in a few days.

Microbial PPPs are selected to have high specificity to the insect pest but also have a number of advantages, including lack of toxic residues, shorter pre-harvest and re-entry intervals for workers, and the potential for a certain amount of self-sustaining secondary control through reproduction and spread within the host population^9^. Microbial PPPs can be applied using conventional spray equipment and benefit from stable, controlled conditions within the greenhouse environment, such as favourable temperatures, partial protection from damaging ultraviolet radiation, and protection from run-off caused by rainfall^10^. However, compared to synthetic chemical pesticides, microbial control agents can be slower-acting, less efficacious, more expensive to purchase, and require tailored training for users^11^.

Few studies have investigated the combination of EPF and chemical pesticides against *T. vaporariorum*^12^. But, in principle, there are good reasons why mixtures of microbial PPPs and synthetic chemical PPPs could present an attractive alternative pest management option. Because conventional pesticides and microbials have different modes of action, there may be synergistic interactions between them that increase the overall level of pest control^13^. A combination treatment may also allow the control of multiple pests or enable pest control over a broader range of environmental conditions^14^. Some studies have shown that microbial PPPs can reduce the chances of resistance developing to a chemical pesticide or as a way of reducing the severity of resistance after it has evolved^15–17^.

It has been suggested that combinations of insect growth regulators (IGR) and EPF can result in synergism due to the prevention of moulting by the insect and the subsequent increase in time for the EPF to penetrate the insect cuticle^18,19^. Due to its high efficacy and low non target effects, the IGR spiromesifen, has been incorporated into many crop protection programmes, especially in protected crops in Spain^20^. This insecticide is sold for control of whitefly and mites in fields and protected crops and prevents moulting and further development of immature stages. However, growers have recently noticed a decrease in the efficacy of the insecticide and reported resistant populations of the Silverleaf whitefly (*Bemisia tabaci*)^20^. In *T. vaporariorum* populations, resistance to spiromesifen has also been detected, although the level of resistance found (in the UK and Europe) was deemed insufficient to reduce the suitability of spiromesifen to control *T. vaporariorum* populations at the time of this study^21^. Establishing a method to improve efficacy and delay resistance development is required to ensure the longevity of this active ingredient.

To establish successful mixtures for pest control, interactions between components in the mixture must first be evaluated and described. In toxicology, mixture concentration–response analysis can be used to establish the joint effect of two or more chemicals at a range of concentrations^22^ which presents a useful framework in which to understand microbial-chemical interactions resulting in additivity, synergism or antagonism, as defined in Supplementary Table S1 online^23^. Toxicologists and ecotoxicologists have been investigating the interactions of multiple chemicals in a range of organisms for nearly a century^24^, and only recently have studies of interacting mixture components been investigated by terrestrial ecologists, for example, the impact on ecosystem services or the potential exploitation of synergistic interactions for pest control^25–29^. The MixTox analysis^23^ allows interactions of co-applied toxicants to be determined and the variation of interactions across data sets to be categorised. Though this analysis has not yet been utilised in insect pathology, the MixTox analysis has the potential to identify interactions between plant protection products which could be exploited for increased pest control.

A series of laboratory experiments were previously conducted to identify EPF candidates with the potential to control *T. vaporariorum* by choosing those that met several selection criteria. The selection criteria used to identify the EPF isolates used in this experiment were related to lethal concentration and dose response, speed of kill, sensitivity to abiotic factors as well as the compatibility of EPF with chemicals used in IPM^25^. Two different species of entomopathogenic fungi were selected, namely, *Beauveria bassiana* and *Cordyceps farinosa.*

In this study, we determined the applicability of the ecotoxicological MixTox analysis method to describe the relationship between co-applied microbial and chemical PPPs using 12- and 14-day mesocosm laboratory bioassays, The primary objective was to characterise interactions between varied lethal concentrations (LC_x_) of EPF and the chemical PPP, Oberon® (active ingredient. spiromesifen), whilst also identifying distinctions in these interactions when different species of entomopathogenic fungi were applied, namely, *B. bassiana* and *C. farinosa*.

## Results

### Combined application of Cordyceps farinosa and spiromesifen against *Trialeurodes* vaporariorum

After 14d, single applications of LC_15_–LC_80_ of *C. farinosa* resulted in *T. vaporariorum* mortality between 5 and 87%, with increasing application concentration resulting in increased mortality. Similarly, a dose–response was observed for increasing concentrations of spiromesifen (LC_15_-LC_80_ ) with *T. vaporariorum* mortality ranging from 7 to 86%. A two-way ANOVA showed that there were significant differences between corrected mortality observed between treatments (F = 8.53, df = 12, p < 0.001) and also across bioassays (F = 3.66, df = 2, p = 0.029). There was no significant difference in conidia received per unit area for each treatment during each bioassay (p = 0.83; Table 1). A post hoc pairwise t-test (p values adjusted for multiple comparisons by Bonferroni method (Bonferroni, 1936) showed that mortality in the third bioassay was significantly different from the second bioassay (p = 0.029), though the third was not significantly different from the first bioassay (p = 0.12), nor were there any differences between bioassay one and two (p = 0.81). Total mortality for bioassays that were significantly different (1&2, 1&3) were analysed separately. Control mortality was 2.7%, 14.3%, and 3.3% in each replicate mixture bioassay involving *C. farinosa* indicating the robustness of the bioassay setup.

**Table 1:** Average dose received on 22 × 22 mm coverslips during spray applications of lethal concentrations (LC) of *Cordyceps farinosa*, spiromesifen or simultaneous applications of both control agents in three replicate mixture bioassays.

Treatment		Dose received (conidia mm^−2^) ± SD		
Spiromesifen	*C. farinosa*	Mixture 1	Mixture 2	Mixture 3
0	LC_15_	11 ± 0.74	12 ± 0.7	10 ± 1
0	LC_50_	83 ± 14	123 ± 5.9	110 ± 6.9
0	LC_80_	731 ± 44	716 ± 126	882 ± 177
LC_15_	LC_15_	12 ± 1.4	14 ± 0.85	10 ± 2
LC_15_	LC_50_	106 ± 30	118 ± 10.31	101 ± 18
LC_15_	LC_80_	1047 ± 98	1085 ± 72	1105 ± 137
LC_50_	LC_15_	11 ± 1.4	15 ± 0.66	11 ± 2
LC_50_	LC_50_	NA	126 ± 11	123 ± 5.4
LC_80_	LC_15_	9 ± 1.35	10 ± 1.84	8 ± 1.57

**Figure 1:**
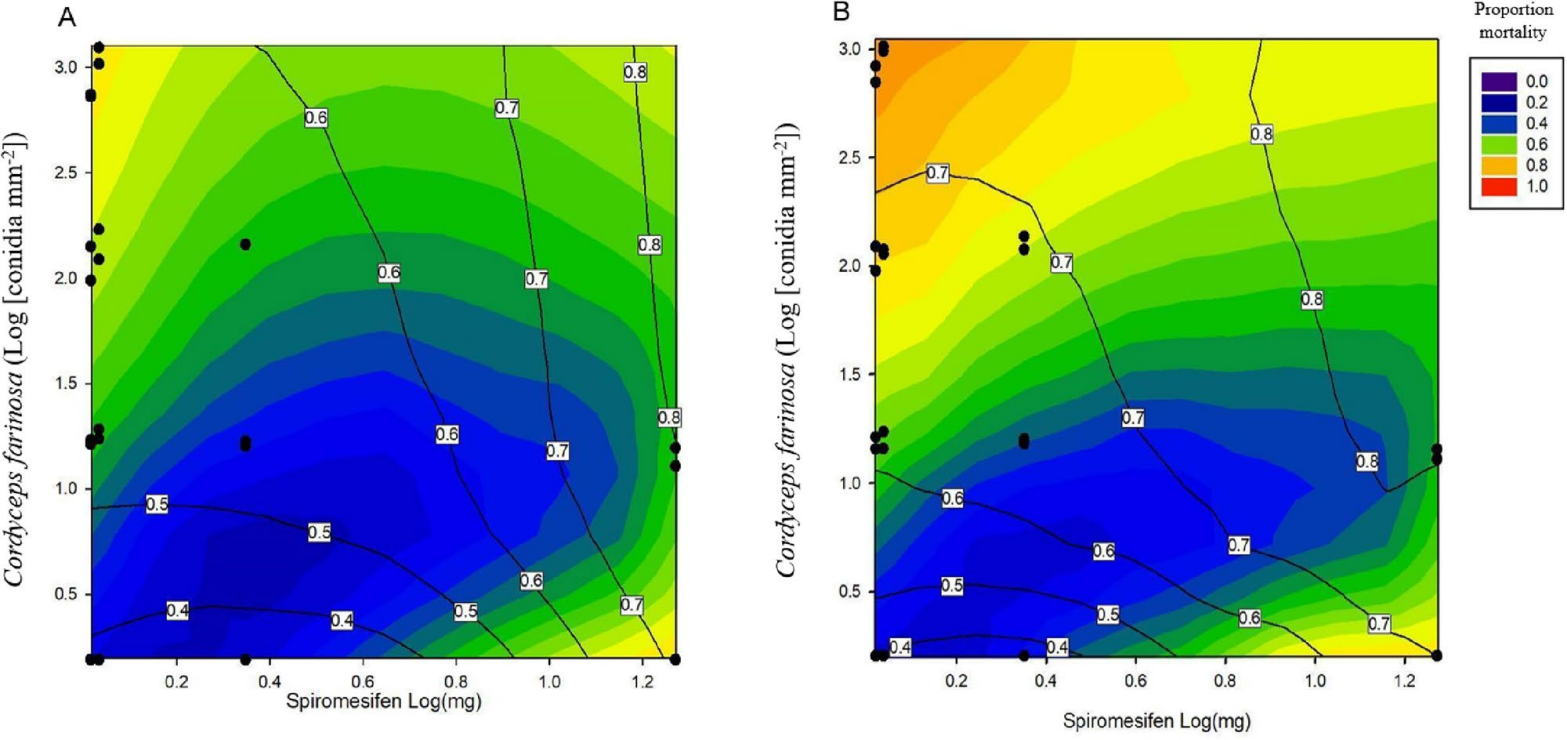
Mixture interactions 14 days after the simultaneous application of *Cordyceps farinosa* and spiromesifen (mg a.i.) across a range of concentration combinations against third instar *Trialeurodes vaporariorum*. In (**A**) proportion mortality was combined across bioassays 1 and 2, whereas (**B**) shows results from bioassays 1 and 3. Observed mortality is displayed by the coloured contours. Expected (additive) proportion mortality based on the effect of each mixture component applied individually is shown as black isobols. Deviation of the contours from the isobols indicates a mixture interaction. Black points overlaid on the plot indicate the dose combinations tested in the experiment.

Synergism, antagonism, and additivity were observed in co-applications of *C. farinosa* and spiromesifen against *T. vaporariorum*. A dose-ratio (DR) model provided the best significant fit for the data from the first two mixture bioassays (R^2^ = 0.57, p < 0.001). The model and parameter values (a = 0.1) indicated that there was antagonism across mixture combinations where the toxicity of the mixture was caused mainly by spiromesifen. However, there was a switch to synergism at low concentrations of spiromesifen when the toxicity of the mixture was mostly caused by *C. farinosa*. The DR model predicted that this switch occurred when the toxicity of spiromesifen was 1.07 × 10^–3^ times the concentration of *C. farinosa* and indicates that spiromesifen was 1.07 times more toxic than *C. farinosa* (see Fig. 1).

When applying the MixTox analysis to separately analysed bioassays, the DR model also provided the best fit for the data (p < 0.001) and described a similar proportion of the variation about the mean (R^2^ = 0.57). The model parameters for bioassays one and three were slightly different than those in the model describing bioassays one and two. There was antagonism for all mixture combinations except where the toxicity of *C. farinosa* was greater than that of spiromesifen. In this model, the switch from antagonism to synergism occurred when the concentration of spiromesifen was 1.2 × 10^–3^ times the effective concentration of *C. farinosa* (see Fig. 1).

Increasing application concentration in single applications of *C. farinosa* or spiromesifen reduced LT_50_ estimates, as shown in Table 2. However, LT_50_ values were not significantly reduced following the application of mixture treatments compared to single treatments of spiromesifen or *C. farinosa.* The four-parameter model significantly fitted the data better than other models tested (p = 0.76) due to significant differences between treatments. Therefore, constraining the LT_50_ value across all treatments did not improve the fit of the model (p = 0.031).

**Table 2:** Total corrected mortality observed by the end of the bioassay and LT_50_ values following the application of *Cordyceps farinosa*, spiromesifen or a combination of these against third instar *Trialeurodes vaporariorum* in laboratory-based experiments.

Lethal concentration of *C. farinosa* (LC)	Lethal concentration of spiromesifen (LC)	Corrected mortality (proportion treated)			LT_50_ (days)		
		Bioassay 1	Bioassay 2	Bioassay 3	Bioassay 1	Bioassay 2	Bioassay 3
LC_15_	0	0.07 ± 0.14	0.05 ± 0.04	0.11 ± 0.032	19.11 ± 1.67	48.32 ± 52.56	19.01 ± 2.59
LC_50_	0	0.24 ± 0.26	0.31 ± 0.16	0.5 ± 0.036	16.27 ± 1.39	20.61 ± 6.54	12.01 ± 0.53
LC_80_	0	0.37 ± 0.18	0.62 ± 0.08	0.87 ± 0.045	15.68 ± 1.69	10.27 ± 1.59	9.38 ± 0.50
0	LC_15_	0.07 ± 0.015	0.27 ± 0.21	0.12 ± 0.2	20.29 ± 2.67	16.94 ± 2.51	18.22 ± 3.25
0	LC_50_	0.74 ± 0.13	0.65 ± 0.05	0.83 ± 0.012	9.75 ± 0.56	10.80 ± 0.84	8.42 ± 0.56
0	LC_80_	0.86 ± 0.06	0.73 ± 0.11	0.84 ± 0.058	8.72 ± 0.46	10.74 ± 1.05	9.23 ± 0.65
LC_15_	LC_15_	0.75 ± 0.32	0.35 ± 0.21	0.45 ± 0	9.53 ± 0.72	16.31 ± 1.68	13.70 ± 0.88
LC_15_	LC_50_	0.15 ± 0.06	0.66 ± 0.21	0.57 ± 0.15	18.95 ± 1.75	10.90 ± 1.59	10.64 ± 1.17
LC_15_	LC_80_	0.81 ± 0.27	0.74 ± 0.11	0.76 ± 0.19	9.19 ± 0.68	12.59 ± 1.58	7.48 ± 1.20
LC_50_	LC_15_	0.91 ± 0.08	0.40 ± 0.11	0.93 ± 0.091	18.95 ± 1.75	10.90 ± 1.59	10.64 ± 1.17
LC_50_	LC_50_	0.13 ± 0.09	0.67 ± 0.04	0.75 ± 0.35	18.93 ± 1.58	10.45 ± 0.50	9.21 ± 0.88
LC_80_	LC_15_	0.65 ± 0.29	0.33 ± 0.34	0.66 ± 0.20	10.31 ± 0.87	11.36 ± 1.33	9.45 ± 1.19

Mortality was corrected using the Schneider–Orelli approach. LT_50_ values were calculated by probit analysis.

### Combined application of Beauveria bassiana and spiromesifen against *Trialeurodes vaporariorum*

**Figure 2:**
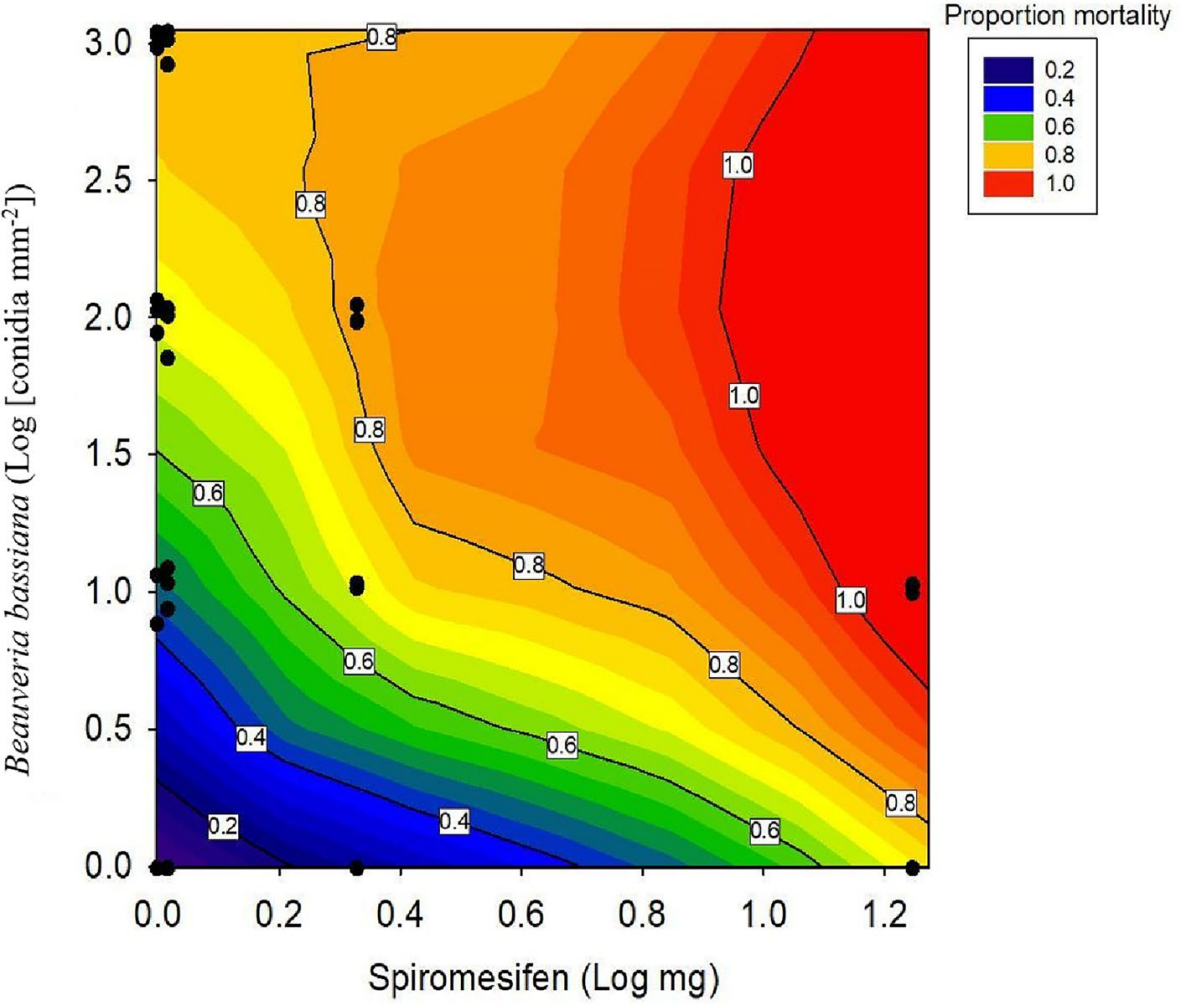
Mixture interactions 14 days after the simultaneous application of *Beauveria bassiana* and spiromesifen (mg a.i.) across a range of concentration combinations against third instar *Trialeurodes vaporariorum*. Proportion mortality was combined across three bioassays. Observed mortality is displayed by the coloured contours. Expected (additive) proportion mortality based on the effect of each mixture component applied individually is shown as black isobols. Deviation of the contours from the isobols indicates a mixture interaction. Black points overlaid on the plot indicate the dose combinations tested in the experiment.

There were significant differences between the total mortality observed at the end of the 12d bioassay for each treatment in the mixture bioassays involving *B. bassiana* and spiromesifen (F = 16.36, df = 12, p =  < 0.001). In single-application treatments, mortality ranged from 2 to 90% depending on the concentration of EPF or spiromesifen applied, with increasing application concentration resulting in increasing mortality. There were no significant differences between mortality for each treatment across bioassays (F = 2.35, df = 2, p = 0.10) or conidia deposition for the application of the same EPF concentration (Table 3). Therefore, further analysis was conducted with data compiled as one dataset across all bioassays. Control mortality was 3.5%, 3.2%, and 10.6% in each bioassay respectively. Total corrected mortality ranged from 0.2 to 88%, depending on the treatment applied. For combined applications, mortality data were successfully described by the Independent action (IA) model (R^2^ = 0.63, p < 0.001), and the addition of parameters to allow for antagonism or synergism did not improve the fit (p = 0.73). Therefore, all mixture outcomes for concentrations applied of *B. bassiana* and spiromesifen resulted in additivity, whereby the observed mortality was not significantly different from the expected mortality based on the single dose response of each component, assuming they follow independent action, as shown in Fig. 2.

**Table 3:** Average dose received by 22 × 22 mm coverslips during spray applications of lethal concentrations (LC) of *Beauveria bassiana*, spiromesifen or simultaneous applications of both control agents in three replicate mixture bioassays.

Treatment		Dose received (conidia mm^−2^) ± SD		
Spiromesifen	*B. bassiana*	Mixture 1	Mixture 2	Mixture 3
0	LC_15_	7 ± 0.85	10 ± 1.81	9 ± 1.2
0	LC_50_	88 ± 4.9	117 ± 8.9	107 ± 20
0	LC_80_	976 ± 69	1114 ± 106	1081 ± 189
LC_15_	LC_15_	8 ± 0.99	11 ± 0.37	10 ± 0.42
LC_15_	LC_50_	71 ± 4.6	102 ± 22	109 ± 14
LC_15_	LC_80_	849 ± 156	1047 ± 199	1117 ± 163
LC_50_	LC_15_	10 ± 1.0	10 ± 1.4	10 ± 1.9
LC_50_	LC_50_	97 ± 7.5	98 ± 14	112 ± 14
LC_80_	LC_15_	9 ± 1.1	10 ± 2	10 ± 0.84

Mortality over the duration of the bioassay was significantly different for treatment (df = 12, F = 11.27, P < 0.001) and time point (df = 7, F = 448.1, p < 0.001), though there was no difference between the mortality over time for the three replicate bioassays (df = 2, F = 2.12, p = 0.12). Total observed mortality did not significantly deviate from the calculated expected mortality. Therefore, particular interest was taken to investigate if LT_50_ values were significantly different for mixture treatments compared to single applications in each bioassay.

Time to kill analysis was performed using probit linear regression. The four-parameter model gave a significantly better fit to the data compared to other models tested (p = 0.87). Constraining the LT_50_ value across all treatments did not improve the fit of the model due to differences between predicted LT_50_ values for different treatments (p < 0.01). Increasing concentrations of *B. bassiana* and spiromesifen applied singly resulted in reduced LT_50_ estimates, except in the second bioassay, where the two highest concentrations of spiromesifen resulted in similar rates of mortality, as seen in Table 4. Though a mixture of LC_50_ *B. bassiana* and LC_50_ spiromesifen did not cause mortality significantly higher than expected, the LT_50_ of this combination was the lowest across all treatments in every repeat of the bioassay, reducing the time to 50% *T. vaporariorum* mortality by up to 5 days compared to applications of LC_50_ of either mixture component alone.

**Table 4:** Total corrected mortality observed by the end of the bioassay and LT_50_ values following the application of *Beauveria bassiana*, spiromesifen or a combination of these against third instar *Trialeurodes vaporariorum* in laboratory based experiments.

Concentration of *B .bassiana*	Concentration of spiromesifen	Corrected mortality (proportion treated) ± SD			LT_50_ (days) ± SE		
		Bioassay 1	Bioassay 2	Bioassay 3	Bioassay 1	Bioassay 2	Bioassay 3
LC_15_	0	0.13 ± 0.10	0.016 ± 0.023	NA	16.07 ± 0.55	20.65 ± 2.84	NA
LC_50_	0	0.49 ± 0.47	0.39 ± 0.23	0.48 ± 0.18	12.51 ± 1.49	13.42 ± 0.82	10.09 ± 0.96
LC_80_	0	0.56 ± 0.05	0.59 ± 0.12	0.9 ± 0.10	10.5 ± 0.9	9.90 ± 0.48	6.51 ± 0.57
0	LC_15_	0.19 ± 0.03	0.12 ± 0.2	0.14 ± 0	18.77 ± 4.16	18.22 ± 3.21	9.02 ± 1.16
0	LC_50_	0.53 ± 0.094	0.83 ± 0.012	0.61 ± 0.002	12.11 ± 0.61	8.42 ± 0.56	10.02 ± 0.51
0	LC_80_	0.78 ± 0	0.84 ± 0.058	0.79 ± 0.03	9.67 ± 0.52	9.23 ± 0.65	7.31 ± 0.61
LC_15_	LC_15_	0.19 ± 0	0.002 ± 0	0.23 ± 0.39	17.86 ± 1.8	16.1 ± 1.63	14.81 ± 3.85
LC_15_	LC_50_	0.73 ± 0.06	0.51 ± 0.31	0.34 ± 0	8.56 ± 0.59	8.97 ± 0.63	12.6 ± 1.14
LC_15_	LC_80_	0.57 ± 0.12	0.81 ± 0.19	0.74 ± 0.15	10.8 ± 0.51	8.84 ± 0.68	7.79 ± 0.59
LC_50_	LC_15_	0.59 ± 0.27	0.29 ± 0.06	0.50 ± 0.29	12.65 ± 1.28	14.36 ± 1.71	9.59 ± 1.11
LC_50_	LC_50_	0.80 ± 0.21	0.85 ± 0.06	0.88 ± 0.12	8.22 ± 0.62	8.64 ± 0.49	6.28 ± 0.56
LC_80_	LC_15_	0.56 ± 0.26	0.56 ± 0.14	0.48 ± 0.16	12.84 ± 0.64	9.17 ± 0.8	14.39 ± 1.12

Mortality was corrected using the Schneider–Orelli approach. LT_50_ values were calculated by probit analysis.

## Discussion

In this study, we demonstrate that spiromesifen mixtures with *C. arinose* resulted in either synergistic or antagonistic activity against *T. vaporariorum* nymphs*,* depending on the ratio of components in the mixture applied. We used the MixTox analysis^23^ to quantify the interactions between the EPFs and the chemical; whilst this approach is commonly used to determine chemical interactions in ecotoxicology, it has not been applied previously to EPF and chemicals although use of this model to study interactions between chemicals and microbial pesticides has been previously proposed by our laboratory^23^. Synergism was found for applications of LC_80_ and LC_50_ of *C. arinose* with LC_15_ spiromesifen. Increasing the relative concentration of spiromesifen resulted in additivity or even antagonism. These results are similar to previous findings that co-application of low concentrations of insecticides with entomopathogenic fungi may increase mortality of some pest species. For example, Santos et al.^29^ found that co-application of a *C. javanica* strain with sub lethal concentrations of spiromesifen resulted in additive or synergistic effects against *B. tabaci* nymphs depending on the concentration applied. Similarly, the combined effect of *B. bassiana* and azadirachtin on third instar *T. vaporariorum* nymphs was investigated by Wei^30^, whereby five mixture treatments of *B. bassiana* and azadirachtin with different ratio combinations were tested. Antagonism was observed with high relative doses of azadirachtin or *B. bassiana* and synergism was found when the ratio was 1:1 or 1:4 (azadirachtin: *B. bassiana*). The dose ratio relationship in the current study suggests that spiromesifen had a negative effect on *C. arinose* when it was the most toxic component in the mixture.

There were no interactions influencing total *T. vaporariorum* mortality following the co-application of *B. bassiana* and spiromesifen. However, co-application of *B. bassiana* and spiromesifen reduced LT_50_ estimates by up to 5 days, depending on the combination applied. A significant increase in the speed of kill as a result of the mixture treatment, despite no change in overall mortality, is beneficial as it reduces the amount of time that pests are on the crop causing damage. Similarly, Ye et al*.*^31^ found that simultaneous application of imidacloprid at a concentration of 0.1–0.5 µg mL^−1^ with *B. bassiana* against the aphid *Myzus persicae* resulted in an increased rate of kill. In another study, Kpindou et al*.*^32^ combined *Metarhizium anisopliae* with lambda-cyhalothrin against Sahelian grasshoppers (*Oedaleus senegalensis*) resulting in EPF –induced mortality occurring as early as 2 days after application, significantly faster than EPF treatments with EPF applied alone. There were no significant differences in the conidial deposition or viability of conidia between singly applied EPF and EPF mixture treatments of the same concentration. Inclusion of the calibration method by Spence et al.^33^ indicates that differences in *T. vaporariorum* mortality were due to mixture interactions rather than differences in conidial deposition between singly applied EPF and mixture treatments. The conidial deposition data was also essential to allow mortality data to be modelled against dose received rather than estimated application concentrations, which may vary between bioassays. Based on the laboratory results demonstrating additivity across all combinations of *B. bassiana* and spiromesifen, there could be potential for this combination to be used in an IPM programme. Other studies support these findings; for example there is evidence that applying *B. bassiana* in combination with azadirachtin, acetamiprid, flonicamid, bifenthrin and avermectin can effectively suppress *T. vaporariorum* populations in strawberry crops, whilst reducing reliance on chemical insecticides^34^. However, further investigations into the effect of abiotic conditions and ultimately, whether additivity between *B. bassiana* and spiromesifen occurs under greenhouse conditions would need to be conducted. In general, the application of synthetic chemicals at low or sublethal concentrations is not recommended as it may lead to increased risk of development of resistance^35^. But, in this case, the application of multiple insecticidal components with different modes of action, especially in combination with other parts of IPM, are likely to reduce development of resistance^36^. Further studies into the susceptibility of *T. vaporariorum* to spiromesifen after repeated exposure to EPF and spiromesifen mixtures over multiple generations could determine whether this approach reduces the evolution of resistance in the population.

In some combinations of *C. farinose* and spiromesifen, synergism was observed, meaning that a higher proportion mortality of *T. vaporariorum* occurred than expected based on mortality caused by these mixture components separately^23^. Despite synergism being observed, antagonism and additivity was also observed between *C. farinose* and spiromesfen observed, meaning that effective control of *T. vaporariorum* could only be assured if a precise ratio of mixture components was delivered to the target pest. In practice, this is difficult to achieve. Several mixture concentrations of each mixture component were investigated because variation in dose received by individual pests within a crop following the application of one concentration is unavoidable. Spatial, temporal, and environmental variation within the crop causes variation in dose of each component in a mixture reaching target insects. The outcome of a mixture depends on the relative doses of the components at the site of interaction. Ideally, the type of interaction occurring between mixture components would have little variation depending on concentration or ratio applied to allow for predictable levels of pest control.

It is well reported in the wider literature that IGRs that prevent or reduce the insects’ ability to develop are thought to improve control efficacy of EPFs^36–38^. Spiromesifen is an IGR that disrupts insect development through the inhibition of lipid synthesis^39^. Shedding EPF conidia by moulting before conidia have penetrated the cuticle is an effective way to avoid EPF infection by insect hosts^40^. If development time between instars is delayed, EPF hase a greater period of time in which to penetrate and infect the host^18^. Whilst the mechanisms behind interactions observed in this study were not investigated, it would be useful to conduct further experiments into the effect of low concentrations of spiromesifen on the development time of *T. vaporariorum*, to indicate whether an increased infection period for the pathogen is the mechanism determining synergism between *C. farinose* and spiromesifen in this study. However, an increased infection period would also be beneficial for the infection of *T. vaporariorum* by *B. bassiana*. Due to the different interactions observed between spiromesifen and *B. bassiana* or *C. farinose*, it is likely that the mechanism behind the interactions observed involves EPF specific compounds (such as species specific secondary metabolites) produced by the EPF or by the host in response to infection. Other mechanisms of synergism have been established in previous studies and are associated with increased stress, immunocompromised hosts and resultant changes in host physiology or behaviour^41^. For example, synergism discovered during the application of sub-lethal concentrations of imidacloprid and *B. bassiana* to second instar coleopteran *Leptinotarsa decemlineata* larvae was determined to be caused by starvation stress which increased larval susceptibility to the pathogen^15^. In another study, the mechanisms behind synergistic interactions of *M. anisopliae* and low concentrations (0.01–0.025 mg/L) of chlorantraniliprole against *Locusta migratoria* were investigated^42^. Activity of glutathione-*S*-transferase, general esterases and phenol oxidase (important detoxifying enzymes) was reduced following co-application of the insecticide and the EPF. The authors speculate that metabolites produced by *M. anisopliae* prevent the activation of detoxifying enzymes by Ca^2+^ disruption, thereby increasing the host’s susceptibility to the insecticide^42^. In a study by Ali et al.^27^, the synergistic interaction of the chemical matrine and *Akanthomyces muscarium* against *B. tabaci* was determined to be related to both matrine and the EPF secondary metabolite, bassianolide, binding to acetylcholine receptors, causing decreased activity of acetylcholinesterase. In addition, co-application of *A. muscarium* and matrine caused an overall reduction in the activity of carboxylesterases (CarE) and glutathione-S-transferase, host enzymes essential for the detoxification of insecticides and pathogens^27^. Therefore, it is evident that mechanisms behind successful mixture outcomes (i.e. synergistic or additive) vary greatly between mixture combinations and the determination of patterns across these studies could aid in the discovery of other effective IPM strategies or allow for further improvements to be made based on the modes of action.

In this study, mixture concentration combinations were chosen based on expected mortality values that did not exceed > 90% assuming independent action, which meant a full factorial experimental design was not conducted. This was to ensure that all interactions, whether resulting in increased (synergism) or decreased (antagonism) mortality could be detected. Several of the previous mixture studies in insect pathology have not used this approach, resulting in redundant treatment groups. For example, a study by Russell et al.^43^ investigated the effect of combining applications of imidacloprid and *M. brunneum* to control Asian long horned beetles (*Anoplophora glabripennis*). However, because every beetle exposed to *M. brunneum* died before the end of the bioassay, interactions were unable to be determined. Another example^12^ saw three concentrations of imidacloprid being simultaneously applied with *B. bassiana* or *C. fumosoroseus* (then taxonomically described as *Paecilomyces fumosoroseus*) on lettuce in greenhouse experiments. The authors reported that low-rate applications of imidacloprid combined with both EPF resulted in increased infection rate and mortality of *T. vaporariorum*. However, the expected mortality in the combined treatments was calculated by the current authors as > 90%, and therefore did not allow significant increases in mortality caused by synergistic interactions to be identified.

This study has shown that quantification of the effects of a mixture involving a microbial pathogen is possible using standardised approaches established in ecotoxicology^26,44^. Though abiotic conditions^45–47^, conidial viability^48^ and host susceptibility influence EPF virulence^49,50^, the rate of the infection process of the insect by the microbial pathogen is ultimately determined by the slowest step i.e. a single rate-limiting enzyme reaction within the EPF^51^. The rate of many biological processes are calculated under this assumption^52^. Therefore, it is reasonable to use the same concentration effect model used for chemicals as a simplification of the behaviour of a microbial pathogen.

Combinations of microbial and chemical PPPs have great potential to be exploited for pest control under IPM^30–32^. But, to improve understanding of mixture interactions, simplistic experiments informed by the theory behind mixture calculations must first be conducted. A priority is to establish the interactions between different elements used within an IPM system, to ensure that one element does not inhibit another. In particular, whether combinations of control agents interact synergistically, antagonistically or give additive effects for selected pests of importance in agriculture and horticulture. Determination of interactions between mixture components is complicated because the type of interactions occurring can vary depending on the concentration of each component^12^, the ratio of components^53^ and the type of application; sequential or simultaneous^54^. In addition, interactions between mixture components may be influenced by biotic factors such as varying susceptibility of different insect stages^55^ and interactions between the target pest and the host plant^56^.

In conclusion, we demonstrate that the co-application of two EPF and the chemical insecticide spiromesifen showed potential for improved control of *T. vaporariorum* in these laboratory experiments. These findings demonstrate the potential for utilising combined applications of entomopathogenic fungi and spiromesifen as an effective integrated pest management strategy. Future studies in the greenhouse will be needed to establish whether these combinations are effective towards a mixed age population of *T. vaporariorum* and under more variable abiotic and biotic conditions. The research presented here, provides a framework for further exploration of mixture interactions and how this may be applied to enhance integrated pest management solutions across agricultural and horticultural settings.

## Materials and methods

### Plant, insect and fungal cultures

Seven-week-old aubergine plants (*Solanum melongena* L., Polemoniales:Solanaceae, var. Paris; Ramiro Arnedo, Spain) were infested with *T. vaporariorum* nymphs following the methods described in^33^. Briefly, this involved the use of purpose-built clip cages that secured 10–20 T*. vaporariorum* adults over a limited area of leaf for 14 h at 24 °C under a 16:8 h light: dark photoperiod. After this period, adults *T. vaporariorum* were removed using a hand-held aspirator. Eggs laid by *T. vaporariorum* adults were left to develop on individual plants in situ inside individual containers for 16 days until they reached the third instar. Individual containers were made from 0.9 L transparent plastic pots (dimensions: height 14.3 cm; width (rim, base) 9.4 cm, 6.7 cm with a circle of nylon mesh for ventilation added to the lid and sealed with formalin; diameter 3.4 cm^33^). At this stage, plants were grouped so that each treatment had at least 70 nymphs spread across three replicate leaves. Target leaves were then sprayed at 138 kPa with 1 mL of either the entomopathogenic fungus *C. arinose* (isolate ATCC 4412) or *B. bassiana* (isolate PPRI 5339), the chemical insecticide Oberon® (spiromesifen 240 g/L; SC 240 Bayer) or a mixture of these. *C. arinose* (isolate ATCC 4412) was sourced from The United States Department of Agriculture Agricultural Research Service collection of entomopathogenic fungal cultures (ARSEF database: https://data.nal.usda.gov) and a commercial sample of *B. bassiana* isolate PPRI5339 was supplied by BASF plc.

### Mixture bioassays

All spray applications were made using a portable mini spray tower which was built and calibrated at the UK Centre for Ecology & Hydrology (Wallingford, UK)^33^. Third instar whitefly nymphs were exposed to concentrations of the microbial or chemical control agents that were expected to cause 15, 50 and 80% mortality (i.e. a lethal concentration or LC_15_, LC_50_ and LC_80_ values, based on preliminary experiments^25^, or mixture treatments involving the combination of *C. arinose* and spiromesifen or *B. bassiana* and spiromesifen. Mixtures involving the LC_15_ for the EPFs or spiromesifen were combined with the LC_15_, LC_50_ and LC_80_ of the second mixture component. Mixtures of LC_80_ with LC_50_ or LC_80_ of each mixture component were not conducted because these combinations would result in 85% and 96% mortality respectively, assuming additivity. It would be difficult to determine positive interactions in these instances as it is likely that all treated *T. vaporarioum* would be dead accounting for some control mortality.

A stock solution of spiromesifen was prepared by adding 75 µl of Oberon® to 100 mL sterile water. Stock suspensions of EPF were prepared following methods described by Spence et al.^33^. These methods involved spreading suspensions of conidia from the first or second subcultures of isolates onto Sabouraud Dextrose Agar (SDA; 65 g per 1 L deionised water), in 90 mm triple-vented plastic petri dishes, sealing with Parafilm™ and incubating at 25 °C for 14 days in the dark. To prepare conidia suspensions for bioassays, 3 ml of Tween 80 in sterile water (0.03% v/v) was applied to culture plates, and conidia were removed by agitating the surface of the dish using a sterile pestle. The suspension was then filtered by pouring the liquid onto double folded sterile muslin cheese cloth and allowed to drip through into a sterile 25 mL tube placed below. This process successfully removed all mycelia and culture debris in the suspension. Vials containing conidial suspensions were then agitated for two minutes by shaking vigorously on a vortex mixer. Concentrations of the resultant stock conidia suspensions were estimated by counting in an Improved Brightline Neubauer haemocytometer (× 400 magnification) and subsequently diluted in sterile 0.03% Tween 80® to give the desired final concentration. Conidia suspensions were kept on ice in an insulated container at 4 °C in the dark for no longer than 24 h before being used in experiments. On the same day as the experimental set up, samples of EPF suspension were observed under the microscope in a haemocytometer to check whether conidia had germinated. No conidia germinated overnight whilst following this procedure.

Solutions of double the target concentration (LC_15_, LC_50_ or LC_80_) were prepared in 50 mL Falcon tubes. For example, if the desired concentration for a treatment of the LC_15_ of *C. farinose* was 1 × 10^4^ conidia mL^−1^, then a stock suspension of 2 × 10^4^ conidia mL^−1^ was prepared. Lethal concentrations of EPF and spiromesifen can be found in Supplementary Table S2 online. By preparing double the required concentration, the mixture treatments were not diluted beyond the desired LC value when combined with the second mixture component. Treatments were prepared in 1 mL LoBind tubes (Eppendorf LoBind® tubes; to reduce conidia binding to tube surfaces) by adding 500 µL of the double concentrate LC stock of either EPF isolate and 500 µL of one of the stock solutions of spiromesifen. Single component treatments were prepared using the same method, except that EPF treatments were combined with 500 µL of Tween 80 (0.03%) and spiromesifen was combined with sterile deionised water.

Dose received per unit area for treatments containing EPF were calibrated using methods described in Spence et al.^33^. This involved spraying individual 22 × 22 mm square glass cover slips with 1 mL of treatment suspension, taken from the same stock solution at the same time as spray applications were made to aubergine leaves. Each treatment was sprayed onto three individual replicate cover slips. Sprayed coverslips were immediately placed individually in 1 mL of 0.03% Tween 80 in 50 mL tubes and serial dilutions were made from 40 μL aliquots taken from each suspension. Diluted suspensions were spread evenly across individual Petri dishes (90 mm diameter) containing 10 mL of SDA. Dishes were sealed with Parafilm and incubated in the dark at 25 °C for 5 days. After that time, the number of colony-forming units (CFUs) were counted and used to calculate the number of conidia received per square millimetre on each coverslip, as an estimate of deposition of conidia on sprayed leaves.

Once solutions applied to the leaf surface had visibly dried (1–2 h), plants were placed in individual plastic containers, as described previously but for the first 24 h following spray application, the lid of the container was sealed in order to maintain high humidity. The containers were incubated at 24 °C with a 16:8 h light:dark photoperiod. After 24 h, container lids were swapped to the ventilated lid previously described. Test leaves in each replicate pot were assessed every 48 h for 14 and 12 days for bioassays involving *C. farinose* and *B. bassiana,* respectively. The instar of surviving nymphs, adult proportion emergence, and mortality were recorded. The bioassay was replicated on three separate occasions.

### Statistical analysis

Mortality at the end of each bioassay was corrected for control mortality using Schneider–Orelli’s equation^57^;

1 $${\text{Corrected mortality }}\left( \% \right) \, = {\text{a}} - {\text{b1}}00 - {\text{b}}*{1}00$$

where *a* is the percentage mortality data from the treated group and *b* is the percentage mortality from the control group.

Differences in total mortality at the end of the bioassay between treatments and between bioassays were determined by ANOVA in R studio (version 4.0.0 2020/04/24).

The predicted combined effect of the two control agents was calculated from the single treatment applications assuming Bliss independence^24^. Under this assumption, each control agent interacting in the mixture kills the target pest by a dissimilar mechanism. This method uses the combination of unaffected fractions to calculate the expected outcome of a mixture.

2 $$Pm=\left(pA\right)(pB)$$

where the probability of an organism surviving the combined treatment of agent A and agent B ($Pm$) would be the probability of an organism surviving agent A ($pA$) multiplied by the probability of an organism surviving agent B ($pB$). Therefore, a mixture consisting of two agents which independently each cause 25% mortality when applied alone will result in 56% mortality as a mixture assuming no interaction between the agents (also known as additivity), by the calculation of (1 − 0.25) * (1 − 0.25) = 1 − survivorship. If the observed mortality is greater than the expected mortality, a synergistic interaction has occurred. Alternatively, antagonism results in lower mortality than expected.

The effect of pathogen—spiromesifen mixtures were determined using the MixTox analysis^23^. Mortality was modeled against dose received for EPF applications in order to account for variation in suspensions applied between replicates and bioassays. The MixTox analysis takes into account the control mortality, so uncorrected data was used for this analysis. In this analysis, a reference model is produced based on mortality observed following experiments to determine the mortality achieved with the single application of each mixture component. The reference model describes the expected outcome of applications of the mixture across a range of concentrations assuming independent action of components based on the single outcomes of the single applications. Mixture effects are characterised based on the deviation of observed mixture data compared to the independent action reference model (i.e., the expected outcomes). Deviations from the independent action model can differ across the model axes. Patterns in the deviation from the reference model can be categorised as absolute synergism/antagonism, dose-level dependent deviation, or dose ratio dependent deviation. Alternatively, if there are no interactions between components in the mixture, there may be no deviation from the reference model. Absolute synergism/antagonism occurs when all concentration combinations result in the same deviation from the reference model, either synergism or antagonism. Dose-level dependent deviation occurs when the deviation differs at a low dose compared to the deviation at a high dose. For example, there may be synergism at low concentrations of both components and antagonism at high concentrations of both components. Dose-ratio dependent deviation occurs when deviation from the reference model is dependent on the proportion of components in the mixture. For example, there may be antagonism when the mixture mainly consists of component 1 and synergism when increasing the proportion of component 2 in the mixture^23^.

Deviations from the reference independent action model are determined by the addition of several parameters to the model and subsequent comparisons between fitted models to determine which describes the data most accurately. The value of each parameter can vary and define the functional form of the deviation pattern. Parameter values and their meaning are shown in Supplementary Table S3.

Estimated time to kill or lethal time to 50% mortality (LT_50_) was calculated using probit analysis in the DRC package in R Studio^58^.

### Ethical approval

*Solanum melongena* L. plants were used in this study. Seeds were purchased from Ramiro Arnedo S.A. Ltd. All methods were performed in accordance with the relevant guidelines/regulations/legislation.

### Supplementary Information

Supplementary Tables.

## Data Availability

The datasets generated during and/or analysed during the current study are available from the corresponding author on reasonable request.
