# Supplementary material for: Improved control of Trialeurodes vaporariorum using mixture combinations of entomopathogenic fungi and the chemical insecticide spiromesifen
Source: Sci Rep. 2024 Jul 3;14:15259. doi: 10.1038/s41598-024-66051-8 (PMC11219850; doi:10.1038/s41598-024-66051-8)
Supplement: Supplementary file 1 — Supplementary Tables. [file 41598_2024_66051_MOESM1_ESM.pdf]

Supplementary information

Definitions of mixture interactions

Supplementary Table S1. Definitions of terms to describe mixture interaction outcomes adapted from Jonker *et al.* 2005.

Term	Definition
Additivity	In a mixture of two or more components, additivity occurs when the observed combined toxic effect is no different from the expected combined effect calculated from single compound toxicities using reference models for non-interaction.
Synergism	the observed combined toxic effect is higher than the expected combined effect calculated from single compound toxicities using reference models for non-interaction.
Antagonism	the observed combined toxic effect is lower than the expected combined effect calculated from single compound toxicities using reference models for non-interaction.

Supplementary Table S2. Lethal concentrations calculated by probit analysis and used in mixture bioassays for combinations of spiromesifen, *Cordyceps farinosa* and *Beauveria bassiana.*

**Lethal Concentration (LC_x)**	**Spiromesifen (mg mL^{-1})**	***C. farinosa* (conidia mL^{-1})**	***B. bassiana* (conidia mL^{-1})**
15	0.04	3.9×10^3	1.6×10^2
50	1.17	2.3×10^6	7.9×10^5
80	17.73	3.5×10^8	7.9×10^8

Supplementary Table S3. Interpretation of parameter values substituted into the MixTox independent action reference model to explain mixture interactions causing observed insect mortality to deviate from the reference model following the application of a mixture. Adapted from (Jonker et al., 2005)

Model type	Parameter	Value	Meaning
Synergism/Antagonism	*a*	>0	Antagonism
		<0	Synergism
Dose ratio dependence	*a*	>0	Antagonism, except for those mixture ratios where significant negative b_i indicate synergism
		<0	Synergism, except for those mixture ratios where significant positive b_i indicate synergism
	b_i	<0	Antagonism where the toxicity of the mixture is mainly caused by toxicant *i*
		>0	Synergism where the toxicity of the mixture is mainly caused by toxicant *i*
Dose level dependence	*a*	<0	Antagonism low dose level and synergism high dose level.
		>0	Synergism low dose level and antagonism high dose level.
	bDL	<2	Change at lower dose level than the EC50*
		=2	Change at the EC50 level
		1< *bDL* <2	Change at higher dose level than the EC50*
		<1	No change, but the magnitude of synergism/antagonism is effect level (IA) dependent

*EC50 = median effect concentration
